# Danhong Injection and Trimetazidine Protect Cardiomyocytes and Enhance Calcium Handling after Myocardial Infarction

**DOI:** 10.1155/2021/2480465

**Published:** 2021-01-15

**Authors:** Jingjing Zhang, Xiaolu Shi, Jinhuan Gao, Rui Zhou, Feifei Guo, Yi Zhang, Fangfang Fan, Qu Zhai, Mingjie Sun, Hongjun Yang

**Affiliations:** ^1^Institute of Chinese Materia Medica, China Academy of Chinese Medical Sciences, Beijing 100700, China; ^2^Beijing Key Laboratory of TCM Basic Research on Prevention and Treatment of Major Disease, Experimental Research Center, China Academy of Chinese Medical Sciences, Beijing 100700, China; ^3^Xuanwu Hospital of Capital Medical University, Beijing 100053, China

## Abstract

Myocardial infarction (MI) is one of the leading causes of death worldwide. However, there is no effective treatment for MI. In this study, trimetazidine (TMZ) and Danhong injection (DHI), representing western medicine and traditional Chinese medicine for MI, were used as tools to identify vital processes in alleviating MI injury. Administration of DHI and TMZ obviously decreased myocardial infarct size, improved ultrasonic heart function, and reduced creatine kinase (CK), lactate dehydrogenase (LDH), and glutamic oxaloacetic transaminase (AST) levels after MI. RNA-seq results indicated calcium ion handling and negative regulation of apoptotic process were vital processes and DHI and TMZ obviously reduced the expression of CaMK II and inhibited cleaved caspase-3 and Bax. Furthermore, DHI and TMZ increased p-S16-PLB, p-S16T17-PLB, CACNA1C, p-RyR2, and p-PKA expression but did not affect SERCA2a expression. In addition to the enhancement of cardiac myocyte shortening amplitude, maximum shortening velocity, and calcium transients, DHI and TMZ increased sarcoplasmic reticulum calcium content and enhanced SERCA2a calcium uptake capability by upregulating the phosphorylation of PLB but did not affect calcium exclusion by NCX. In conclusion, DHI and TMZ protect against MI through inhibiting apoptosis by downregulating CaMKII pathway and enhancing cardiac myocyte contractile functions possibly through the PKA signaling pathway.

## 1. Introduction

Myocardial infarction (MI), caused by a sudden decrease in or interruption of coronary blood supply and oxygen, is a leading cause of death worldwide, resulting in acute and persistent ischemia in the myocardium, followed by myocardial apoptosis and necrosis [1]. A large number of cardiomyocytes die as a result of the deficiency of oxygen and nutrients at the ischemic site, which initiate a complicated pathological process along with inflammation, oxidative stress, and energy insufficiency, and others [2]. The ischemic myocardium is characterized by ruptured myocardial myofibrils, swollen and fractured mitochondria, damaged microvascular structures, extensive inflammation, and elevated levels of reactive oxygen species. Timely reperfusion after MI depending on thrombolytic treatment including primary percutaneous coronary intervention (PPCI) is the only therapy to inhibit irreversible ischemic injury, restore blood flow, and reduce infarct size [3]. However, reperfusion usually exacerbates ischemic damage, resulting in arrhythmia, systolic dysfunction, microvascular disorder, and myocardial cell death [4, 5]. Therefore, it is important to discover cardioprotective therapies for myocardial ischemia.

Western medicine, usually represented by monomer drugs, often acts on specific target with specific effects [6]. In contrast, traditional Chinese medicine (TCM) is a multi-targeted therapy due to its multi-component composition and has become an increasingly important complementary and alternative medicine in clinical practice. In this study, trimetazidine and Danhong injection, which represent typical western medicine and TCM for myocardial infarction, respectively, were used as tools to mechanisms underlying MI and to identify vital processes in alleviating MI injury. Trimetazidine (TMZ) is a typical widely used medicine for cardiovascular diseases [7, 8]. Notably, TMZ can markedly elevate the levels of important antioxidant enzymes such as SOD and GSH-Px and inhibit MDA production (the peroxidation product of lipid) [7, 8]. As a metabolic agent, it exerts a cardioprotective effect by alleviating oxidative stress damage, improving calcium overload, and maintaining ATP levels but does not impact vasodilatory properties or hemodynamic actions [8, 9]. Danhong injection (DHI), derived from *Salvia miltiorrhiza* and *Carthamus tinctorius*, is widely used for cardiovascular diseases and ischemic encephalopathy, including myocardial ischemia [10–13]. It reduces Ca^2+^ overload and free radical production and inhibits subsequent mitochondrial permeability by promoting transitional pore opening in response to hydrogen peroxide- and hypoxia/reoxygenation-induced injury [11, 12]. *In vivo*, a marked reduction in the area of myocardial infarct and the level of myocardial damage-related enzymes such as CK in MI is observed after DHI treatment [13]. In addition, DHI inhibits cardiomyocyte apoptosis, improves angiogenesis, slows myocardial remodelling, and enhances cardiac function after MI [14]. However, the mechanism of DHI and TMZ remains unclear, especially in the early stages of MI. In this study, TMZ and DHI were used as tools due to their effectiveness in treating myocardial ischemia. By taking advantage of RNA-seq technologies, vital biological processes for treating myocardial ischemia were revealed. This study would help to systematically understand the mechanism of MI and facilitate the clinical use of these two drugs.

## 2. Materials and Methods

### 2.1. Animal Model

Adult male Sprague-Dawley rats (270 ± 10 g) were purchased from the Experimental Animal Center of Peking University Health Science Center, Beijing, China [(certificate no. SCXK (Jing) 2009-0017)] and housed in a room with proper temperature and humidity under a 12/12-hour light-dark cycle. All of the experiments were conducted according to the guidelines of the Committee on Animal Care and Use of Institute of Chinese Materia Medica, China Academy of Chinese Medical Sciences. The rats were given free access to water and food and allowed to acclimatize for 7 days before any experiments.

### 2.2. Myocardial Ischemia Surgery and Drug Administration

After anesthesia with pentobarbital sodium (10 mg/ml, ip), myocardial ischemia was induced in animals by tightening a slipknot around the left anterior descending coronary artery with a 6-0 silk suture. The same procedures were performed on sham group rats, but the silk suture was not tied after passing beneath the left anterior descending coronary artery. The rats were randomly divided into six groups: (i) sham; (ii) myocardial infarction (MI, receiving saline); (iii) TMZ + MI (receiving trimetazidine at 10 mg/kg, ip); (iv) D0.42 + MI (receiving DHI at 0.42 ml/kg, ip); (v) D1.05 + MI (receiving DHI at 1.05 ml/kg, ip); and (vi) D2.1 + MI (receiving DHI at 2.1 ml/kg, ip). The dose of TMZ was determined according to previous research [15, 16]. The drugs were administered 24 hours and 10 min before the myocardial ischemia surgery, respectively. Danhong injection is composed of *Salvia miltiorrhiza* and *Carthamus tinctorius* and was provided by Shandong Danhong Pharmaceutical Co., Ltd. (drug approval number: Z20026866).

### 2.3. TTC Staining and Echocardiography

Rat hearts were harvested and sliced into five sections of 1 mm thickness across the left ventricular long axis. To identify the infarct area, the heart sections were incubated with triphenyltetrazolium chloride (TTC, 1% g/ml, Solarbio Technology) for 15 min at room temperature. Infarction rates were measured using Image-J software. The infarction rate is shown as a percentage of the infarct area over the total area. After 24 hours of infarction induction, rats were anesthetized with isoflurane, and echocardiographic measurements were performed. Left ventricular ejection fraction (LVEF) and left ventricular fractional shortening (LVFS) were measured and calculated using Vevo 770 (Visual Sonies Imaging System, Toronto, ON, Canada) and related software through detection along the short axis of the left ventricle at the papillary muscles.

### 2.4. Biochemical Analysis of CK, LDH, and AST Activity

Rat blood samples were collected after 24 hours of ischemia. The kits for creatine kinase (CK, A032), lactate dehydrogenase (LDH, A020-2), and aspartate transaminase (AST, C010-1) were purchased, and signals were detected using a microplate reader (Molecular Devices, USA) using the detection kit purchased from Nanjing Jiancheng (China).

### 2.5. Immunofluorescence Staining

For immunofluorescence staining, samples were fixed with 4% (*v*/*v*) polyformaldehyde and incubated with Triton X-100 (0.5% *v*/*v*) for 30 min before serum blocking for 60 min. Then, antibodies against Bax (ab182733) and Bcl-2 (ab196495) were added for 24 hours at 4°C before incubation with a secondary antibody (1: 200) in the dark at 37°C for 60 min. After washing with PBS, 4, 6-diamidino-2-phenylindole (DAPI; Sigma, USA) was used to stain the nuclei, and photos were taken under a confocal microscope (LSM510; Zeiss). One-Step TUNEL Apoptosis Assay Kit (C1086, Nanjing Jiancheng) was employed to assess myocardial apoptosis through specific detection of DNA breaks in apoptotic cells according to the instructions included in the kit.

### 2.6. Isolation of Ventricular Myocytes

SD rats were grouped and pretreated as described above, and LV myocytes were enzymatically isolated as previously described [17]. In brief, after an SD rat was anesthetized, the heart was removed via sternotomy and placed on the Langendorff perfusion aortic cannula. The heart was retrogradely perfused for 5 min with oxygenated (100% O_2_) normal Tyrode's (NT) solution (concentration in mM: 137.0 NaCl, 1.2 NaH_2_PO_4_, 5.0 KCl, 1.2 MgCl_2_, 10.0 HEPES, 10 glucose, and 1.2 CaCl_2_ (pH 7.4)). Then, the perfusate was switched to Ca^2+^-free Tyrode's solution for 5 min, followed by perfusion for approximately 25 min with the same solution containing 30 *μ*m CaCl_2_ and 0.6 g/ml type II collagenase (Worthington Biochemical). Next, ventricular tissue at the edge of the infarct area was removed (tissue from the same location was collected for the sham group) and minced in KB solution (concentration in mM: KOH, 120; MgCl_2_, 5; L-glutamate, 120; Taurine, 20; HEPES, 10; EGTA, 1; and D-glucose, 10; pH 7.3 with KOH)) to obtain single myocytes. The cells were filtered through a nylon mesh and resuspended in centrifuge tubes for centrifugation (400 rpm for 30 s), and the supernatant was then discarded. The myocytes were resuspended, and extracellular Ca^2+^ was slowly added back up to 1.2 mm. Calcium-tolerant and rod-shaped cells showing clear cross striations were used for Ca^2+^ transient and sarcomere shortening measurements, respectively.

### 2.7. Cytosolic Ca^2+^ Transient and Sarcomere Shortening Measurements

Isolated cardiac myocytes were loaded with 2 *μ*m Fura-2 AM in the dark for 30 min at room temperature, and fluorescence and sarcomere length measurements were recorded with Fluorescence Measurement and Cell Dimensioning Systems (IonOptix, USA). After loading, cells were washed and resuspended twice in NT solution and then placed in a cell chamber, stimulated with a 4 ms duration electrical stimulation at 1 Hz, and superfused at room temperature. Myocytes were exposed to 340 or 380 nm excitation wavelength. The emitted fluorescent signal was detected at 510 nm. The IonWizard software (IonOptix) was used to synchronously record fluorescence and sarcomere length in myocytes. At the end of the recording, the background fluorescence for individual myocytes was detected by moving the view to a nearby blank area. IonWizard was used to correct the Fura-2 AM ratio by subtracting the background and analyzing the calcium transient and sarcomere shortening.

### 2.8. Sarcoplasmic Reticulum Calcium Content and Removal Assessment

The sarcoplasmic reticulum calcium content is related to caffeine-sensitive Ca^2+^ release in response to the rapid application of caffeine. The process to assess sarcoplasmic reticulum calcium content has been described by Bassani et al. [18]. Myocytes were perfused with Tyrode's solution and stimulated at 1 Hz. Rapid and continuous application of 10 mm caffeine was employed to induce SR Ca^2+^ release and assess the contribution of NCX and slow transport systems. Rapid changes in superfusate were achieved using an ALA quick switch system to change the solution bathing the cell, thus allowing us to assess the immediate effect of caffeine on cell contraction. In addition to continuous caffeine superfusate, a decrease in fluorescence (F340/380) indicates calcium removal, which is attributable to NCX and slow transport systems (mitochondrial Ca^2+^ uniporter and sarcolemmal Ca^2+^-ATPase).

### 2.9. Western Blotting

Heart tissues were minced as small as possible, and RIPA lysis buffer (R0020; Solarbio) and protease inhibitor (0.1% phenylmethanesulfonyl fluoride, PMSF; Solarbio) were added to lyse the samples. After the addition of 4× sodium dodecyl sulphate (SDS) loading buffer (P1015; Solarbio, China), the mixture was boiled. Western blotting was conducted according to the manufacturer's protocol. The used antibodies were as follows: anti-SERCA2a ATPase antibody (ab2861), anti-CaMKII; antibody (6G9) (ab22609), antiphospholamban antibody (ab2865), antiphospholamban antibody (phospho-S16) (ab15000), antiphospholamban antibody (phospho-S16 + T17) (ab62170), anti-CACNA1C antibody (ab84814), and anti-GAPDH mouse monoclonal antibody (Proteintech, 60004-1-l g). The antibodies were incubated with the membrane for 24 hours at 4°C, and then horseradish peroxidase-conjugated secondary antibodies were added for 1 hour before detection by ChemiDoc XRS + Molecular Imager (Bio-Rad, USA).

### 2.10. Systematic Investigation of Gene Expression Profiles by the RNA-Seq Technology

According to the instructions, RNA from heart tissue was harvested with TRIzol reagent (Cat# 15596-018, Life Technologies, USA). The RNA Nano 6000 Assay Kit corresponding to the Bioanalyzer 2100 system (Agilent Technologies, CA, USA) was used to analyze the integrity of RNA. One microgram of RNA from each sample was used for subsequent library construction, and each group had three biological replicates. Then, an Illumina® based NEBNext® Ultra™ RNA Library Prep Kit (NEB, USA) was employed to construct sequencing libraries. And the sequencing process was according to previous research [10]. Novogene Bioinformatics Technology Co., Ltd. (Beijing, China) helped to finish library construction and sequencing experiments.

The rat reference genome (Ensemble release 83) was used for read mapping. HTseq v0.6.1 was used to measure the mapped read numbers. The gene expression level was presented by FPKM, which stands for fragments per kilobase of transcript per million mapped reads. The differential gene expression was analyzed using EdgeR software [19]. The raw data of DHI- and TMZ-mediated protection against myocardial ischemia were uploaded into https://dataview.ncbi.nlm.nih.gov/object/PRJNA533004. Gene functional annotation of the obtained gene expression data was further performed using DAVID, and interaction networks were constructed using Cytoscape v3.4.0 [20, 21].

### 2.11. Statistical Analysis

The data obtained from the experiments were analyzed using SPSS v.17.0 software (one-way ANOVA, LSD, *P* < 0.05), and the results are shown as the mean ± standard deviation (SD) with significant *P* values set as ^*∗*^*P* < 0.05 versus sham, ^#^*P* < 0.05 versus MI, and ^Δ^*P* < 0.05 versus D2.1 + MI.

## 3. Results

### 3.1. DHI and TMZ Reduced Myocardial Infarction and Enhanced Heart Function

**Figure 1:**
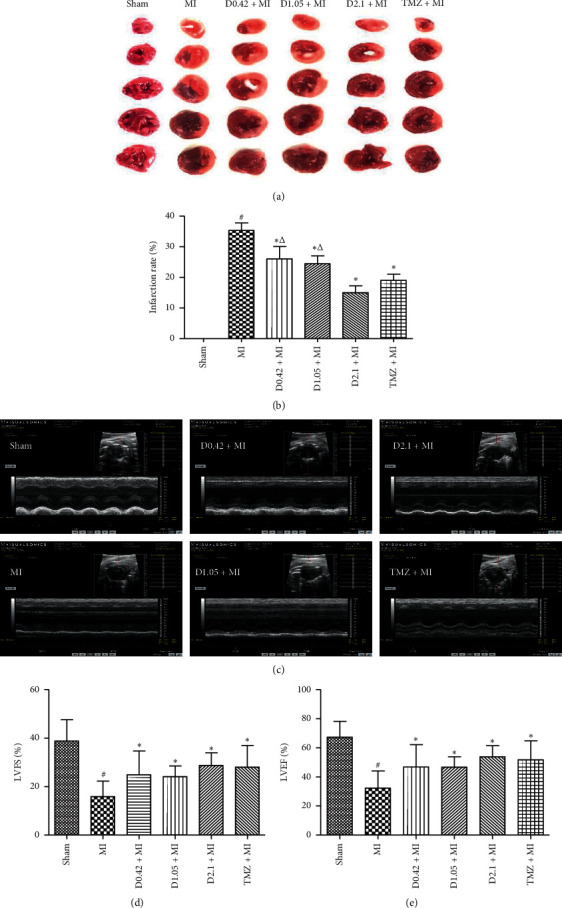
DHI and TMZ treatment reduced myocardial infarction and enhanced heart contractile functions. (a) TTC staining of heart tissues indicated reduction in myocardial infarction by DHI. (b) The statistical analysis of infarction rate in heart tissues as calculated by Image-J software (*n* = 6). (c) Echocardiography images under short-axis M-mode showed distinct heart functions after various treatments. (d) Left ventricular ejection fraction (LVEF) and (e) fractional shortening (FS) were calculated from echocardiography images (*n* = 6). Data are expressed as the mean ± SD, ^#^*P* < 0.05 compared to sham, ^*∗*^*P* < 0.05 compared to MI, and ^Δ^*P* < 0.05 compared to D2.1 + MI.

The pharmacological effect of DHI and TMZ against MI was evaluated by TTC staining and echocardiography. MI operation caused a significant increase in infarct size (Figures 1(a) and 1(b), *P* < 0.05) and a decrease in LVEF and LVFS (Figures 1(c)–1(e), compared to sham operation, *P* < 0.05). However, treatment with DHI or TMZ markedly reduced the infarction rate (compared to MI, *P* < 0.05), and the decrease in the infarction rate induced by DHI was in a dose-dependent manner. Briefly, an obvious decrease of infarction rate was observed in MI treated with DHI at 2.1 ml/kg when compared to MI treated with DHI at 0.42 ml/kg and 1.05 ml/kg (*P* < 0.05). And there was no difference in infarction rate between D2.1 + MI group and TMZ + MI group. In contrast to those by MI alone, LVEF and LVFS were obviously enhanced by DHI and TMZ treatment.

### 3.2. “Negative Regulation of Apoptotic Process” and “Cellular Calcium Ion Homeostasis” Were Identified as Vital Processes in DH- and TMZ-Mediated Protection by RNA-Seq Analysis

**Figure 2:**
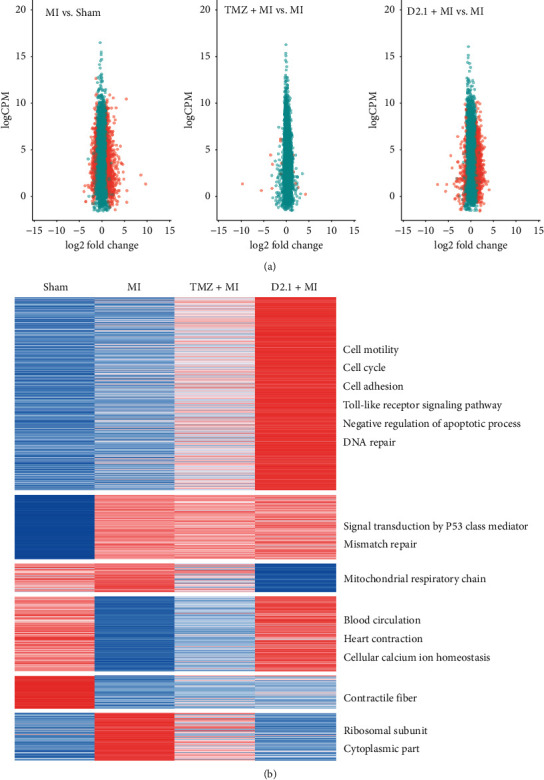
Systematic investigation of the mechanism in DHI- and TMZ-mediated protection by by RNA-seq analysis: (a) alterations in the whole genome shown as a volcano plot. Red dots represent differentially expressed genes, and green dots represent nondifferentially expressed genes (*n* = 3). (b) Differentially expressed genes were clustered as a hierarchical heatmap.

There were 363 significantly downregulated genes and 973 significantly upregulated genes after MI surgery. In contrast, 1497 upregulated genes and 179 downregulated genes were observed in DHI-treated rats. In contrast to that with DHI treatment, only 14 significantly altered genes were found with TMZ treatment (see Table S1). As indicated by Figure 2(b), MI suppressed genes involved in biological processes such as “heart contraction” and “cellular calcium ion homeostasis.” However, these biological processes were slightly reversed by TMZ and further reversed by DHI. Compared to those in the sham group, the genes involved in biological processes such as “negative regulation of apoptotic process,” “DNA repair,” and so on were slightly enhanced in the MI group and further upregulated in the TMZ- and DHI-treated groups.

**Figure 3:**
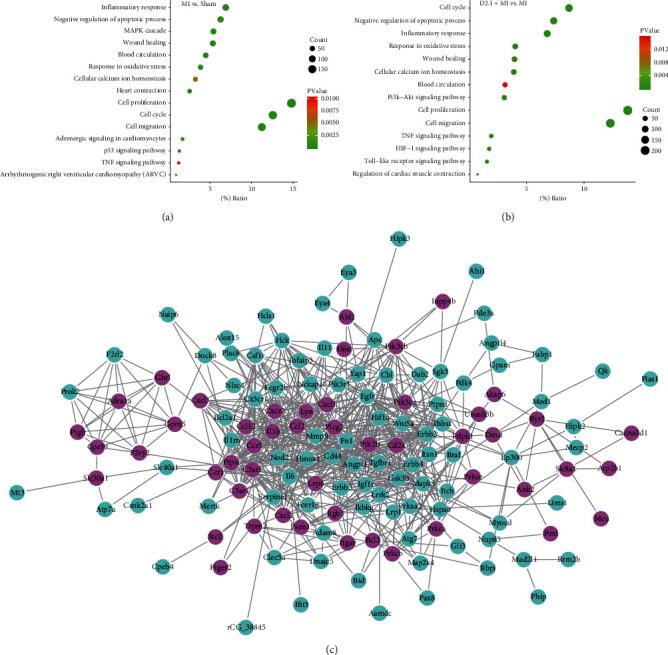
The GO or KEGG pathways enriched by the differentially expressed genes (DEs) in DHI- and TMZ-mediated protection against MI. (a) The GO or KEGG pathways enriched by the DEs after MI surgery. (b) The GO or KEGG pathways enriched by the DEs in MI after DHI treatment. (c) The regulatory network of DEs involved in the terms of “cellular calcium ion homeostasis” and “negative regulation of apoptotic process” after DHI treatment (the DEs involved in “cellular calcium ion homeostasis” are labelled red, and the DEs involved in “negative regulation of apoptotic process” are labelled blue).

The differentially expressed genes (DEs) between various treatments were evaluated in terms of Gene Ontology (GO) or Kyoto Encyclopedia of Genes and Genomes (KEGG) and presented as GO enrichment plots. As indicated in Figure 3, the DEs between the MI and sham groups were enriched in GO or KEGG terms including “cell cycle,” “inflammatory response,” “negative regulation of apoptotic process,” “heart contraction,” “apoptotic process,” “cellular calcium ion homeostasis,” “wound healing,” and “p53 signaling pathway.” In contrast, the DEs between the D2.1 + MI and MI groups were enriched in GO or KEGG terms such as “inflammatory response,” “cellular calcium ion homeostasis,” “negative regulation of apoptotic process,” “regulation of cardiac muscle contraction,” among others. Importantly, the gene ratio of DEs after MI involved in the biological process of “negative regulation of apoptotic process” was 6.28% and went up to 7.37% in DHI-treated group. Additionally, the gene ratio of DEs after MI involved in the biological process of “cellular calcium ion homeostasis” was 3.25% and went up to 3.90% in the DHI-treated group. In contrast to those after DHI treatment, the DEs after TMZ treatment were enriched in biological processes such as “cytoplasm” and “cellular metabolic process” (data not shown). A network of the DEs involved in these two terms was constructed by Cytoscape and a tight network of the DEs involved in these two terms was observed. Therefore, the DEs involved in the biological processes “negative regulation of apoptotic process” and “cellular calcium ion homeostasis” may play a vital role in DHI-mediated protection against MI.

### 3.3. DHI and TMZ Decreased CK, LDH, and AST Levels and Inhibited CaMKII-Related Apoptosis

**Figure 4:**
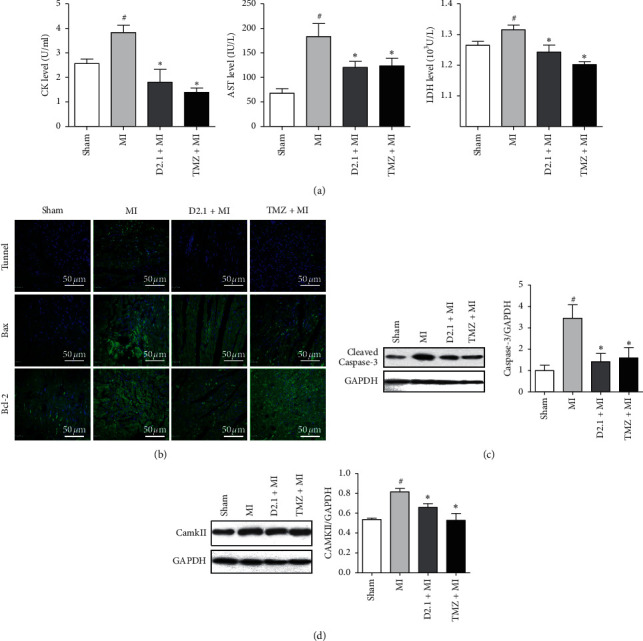
DHI and TMZ reduced myocardium damage, inhbited cell apoptosis, and decreased CaMKII. DHI and TMZ reduced myocardium damage, inhbited cell apoptosis, and decreased CaMKII. (a) Serum CK, AST, and LDH levels (*n* = 6). (b) TUNEL staining and immunofluorescence staining of Bax, Bcl-2; scale bar for TUNEL and Bax and Bcl-2 staining was 50 *μ*m. (c) Representative western blotting for cleaved caspase-3 and its quantitation (*n* = 3). (d) Representative western blotting for CaMKII and its quantitation (*n* = 3). The data are shown as the mean ± SD, ^#^*P* < 0.05 compared to sham, ^*∗*^*P* < 0.05 compared to MI.

As indicated by Figure 4, DHI and TMZ treatment obviously decreased CK, LDH, and AST levels (compared to MI, *P* < 0.05). In addition, MI surgery caused obvious cell apoptosis as evidenced by positive TUNEL staining and increased western blotting results of cleaved caspase-3. In contrast to MI, DHI and TMZ treatment inhibited cellular apoptosis. Increased staining of Bax was reduced after DHI and TMZ treatment while no obvious change of staining of Bcl2 was observed between various treatments. Calcium/calmodulin-dependent protein kinase II (CaMK II) is a central regulator of intracellular Ca^2+^ transport and inducer of cell apoptosis. Thus, we hypothesized that DHI and TMZ might exert an effect on CaMK II to prevent cell apoptosis. The western blotting results showed an obvious increase in CaMK II protein levels in MI, whereas both DHI and TMZ treatment reversed the increase in CaMK II.

### 3.4. DHI and TMZ Enhanced Contractile Functions and Calcium  Transient in Ventricular Myocytes at the Infract Edge

**Figure 5:**
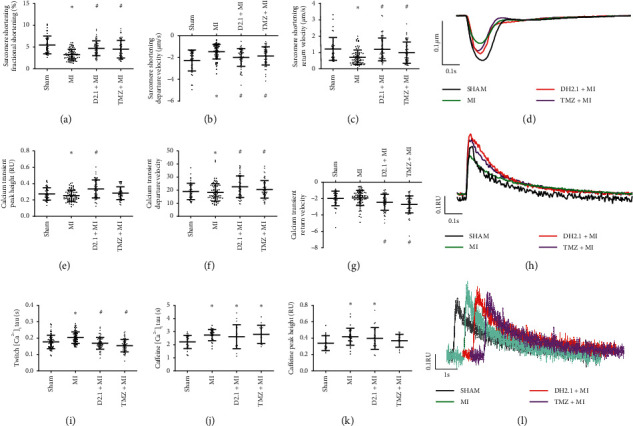
The effect of DHI and TMZ on cardiac myocyte sarcomere shortening and calcium transient at the infracted edge. (a) Sarcomere shortening amplitude; (b) maximum sarcomere shortening velocity; (c) maximum sarcomere relaxation velocity; (d) typical sarcomere shortening curve pacing at 1 Hz; (e) calcium transient amplitude (RU, 340/380 nm fluorescence ratio unit); (f) maximum calcium release velocity in calcium transients; (g) maximum calcium decline velocity in calcium transients; (h) typical calcium transient curve pacing at 1 Hz; (i) twitch-induced calcium decline time constant; (j) caffeine-induced calcium decline time constant; (k) sarcoplasmic reticulum calcium content (caffeine-induced); (l) typical caffeine-induced calcium release curves. The data are shown as the mean ± SD, *n* = 41–47 from 4 hearts for each group, ^#^*P* < 0.05 compared to sham, ^*∗*^*P* < 0.05 compared to MI.

Ventricular myocytes at the infract edge were synchronously analyzed to record calcium transient and sarcomere shortening at 1 Hz pacing. Figures 5(a)–5(c) show that the contractile function of myocytes, depicted by fractional shortening (shortening amplitude, 3.27 ± 1.22% in the MI group vs. 5.49 ± 2.04% in the sham group, *P* < 0.01), departure velocity (maximum shortening velocity, 1.46 ± 0.67 *μ*m/s in the MI group vs. 2.28 ± 0.96 *μ*m/s in the sham group, *P* < 0.01), and return velocity (maximum relaxation velocity, 0.71 ± 0.45 *μ*m/s in the MI group vs. 1.22 ± 0.70 *μ*m/s in the sham group, *P* < 0.01), in the MI group was decreased after myocardial infarction. Compared to the MI group, the DHI-treated group had increased fractional shortening (shortening amplitude, 4.73 ± 1.68%, *P* < 0.01), departure velocity (2.00 ± 0.80 *μ*m/s, *P* < 0.01), and return velocity (1.20 ± 0.70 *μ*m/s, *P* < 0.01) of myocardial cells after myocardial infarction. TMZ had a similar effect as DHI on fractional shortening (shortening amplitude, 4.53 ± 2.04% compared with MI alone, *P* < 0.01), departure velocity (1.85 ± 0.83 *μ*m/s, *P* < 0.01), and return velocity (0.99 ± 0.65 *μ*m/s, *P* < 0.01).

Calcium handling results showed that although the diastolic calcium concentration was slightly increased (non-significant, data not shown), the calcium transient amplitude was decreased (0.253 ± 0.068 RU (ratio unit) in the MI group vs. 0.274 ± 0.075 in the sham group). In the DHI- and TMZ-treated groups, diastolic calcium concentration was slightly increased. However, compared to the MI group, the calcium transient amplitude was increased in the DHI- and TMZ-treated groups at 0.335 ± 0.110 RU and 0.283 ± 0.078 RU, respectively. Corresponding increases were also observed in departure velocity (maximum calcium release velocity in calcium transients) and return velocity (maximum calcium decline velocity) in the DHI- and TMZ-treated groups (Figures 5(e)–5(g)). Importantly, DHI did not affect the amplitude changes of contraction and calcium transient when incubated DHI with the isolated left ventricular myocytes (Supplementary materials (available here)). Compared with TMZ-treated group, the calcium transient amplitude was increased in the DHI-treated group (*P* < 0.05), which indicated that more calcium was released from the sarcoplasmic reticulum in the excitation-contraction coupling.

### 3.5. DHI Altered Twitch- and Caffeine-Induced Calcium Transients

In the caffeine application assessment, the caffeine-induced Ca^2+^ transient deflection time constant (taucaff) indicated the functionality of NCX in removing diastolic Ca^2+^. taucaff was extended in the MI, DHI-treated, and TMZ-treated groups to 2.73 ± 0.43 s, 2.59 ± 0.83 s, and 2.70 ± 0.62 s (compared with the sham group 2.18 ± 0.52 s, *P* < 0.01), respectively. Interestingly, the caffeine challenge results showed that cardiac myocyte sarcoplasmic reticulum calcium content increased after myocardial infarction in the MI and DHI-treated groups (compared with the sham group, *P* < 0.05). The SR calcium content was 0.339 ± 0.091, 0.419 ± 0.103, 0.397 ± 0.133, and 0.369 ± 0.078 for the sham, MI, DHI-treated, and TMZ-treated groups, respectively (Figure 5(k)). The possible mechanism was decreased NCX activity. A decreased NCX activity indicates reduced calcium exclusion from the intracellular space and a more efficient role of SERCA in the decrease in calcium during the diastolic phase. Calcium content and taucaff were not significantly different between the DHI- and TMZ-treated groups. Even though the diastolic calcium concentration was increased compared with that in the sham group, it did not cause effective release of calcium from the sarcoplasmic reticulum. The underlying mechanism may be that RyR activity was decreased after myocardial infarction.

In contrast to that in the MI group, a decrease in tautwitch in the DHI- and TMZ-treated groups indicated an increase in calcium exclusion. On the other hand, the caffeine challenge results showed that taucaff was increased with both DHI and TMZ treatment, similar to that with MI alone, indicating low NCX activity. It can be concluded that SERCA activity was upregulated by DHI to induce calcium uptake by the sarcoplasmic reticulum. Compared with the DHI-treated group, the tautwitch of TMZ-treated was downregulated indicating a stronger calcium removal capability. It could be inferred that TMZ-treated myocytes had higher SERCA activity. To investigate the direct effect of DHI on the isolated left ventricular myocytes, the amplitude changes of contraction and calcium transient before and after drug administration were measured. As indicated in Table S2, it demonstrated that DHI had no effect on the contraction of myocytes directly.

### 3.6. DHI and TMZ Altered Calcium Handling Regulation-Related Protein Expression

**Figure 6:**
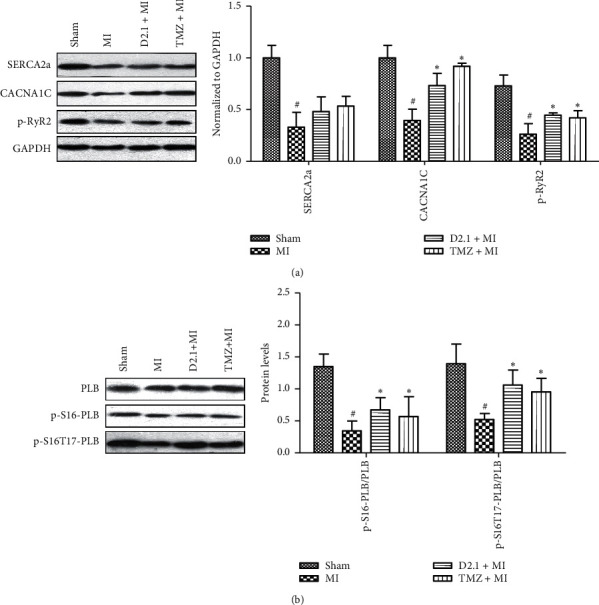
DHI and TMZ improved Ca^2+^ handling-related protein levels in myocardial ischemia in rats. Western blotting results for (a) CACNA1C, SERCA2a, and p-RyR2 (*n* = 3) and (b) PLB, p-S16-PLB, and p-S16T17-PLB (*n* = 3) in ischemic heart tissues. Image-J was used to quantify the protein levels, and the data are shown as the mean ± SD, ^#^*P* < 0.05 compared to sham, ^*∗*^*P* < 0.05 compared to MI.

A significant reduction in CACNA1C protein levels was observed in the MI group. However, both DHI and TMZ treatment significantly increased CACNA1C protein levels compared to MI. The same trend was also observed for the RyR2 ryanodine receptor isoform expression. RyR2 activity was reduced after myocardial infarction (Figure 6) and upregulated after DHI and TMZ treatment. The upregulated RyR activity resulted in more sensitivity to calcium release from the sarcoplasmic reticulum during EC coupling. DHI increased diastolic calcium concentration and calcium transient peak heights compared with MI alone, indicating possible mechanisms by which DHI enhanced cardiac myocyte contractility in response to myocardial infarction. This study also measured the protein levels of SERCA2a, p-S16-PLB, and p-S16T17-PLB, which are mainly affected by calcium uptake into the sarcoplasmic reticulum. The significant decrease in SERCA2a protein levels in response to MI was not alleviated by DHI or TMZ. In contrast to the MI group, p-Ser16-PLB and p-S16T17-PLB were upregulated in the DHI- and TMZ-treated groups (Figure 6). p-PKA expression was also upregulated in the DHI- and TMZ-treated groups.

Notably, DHI and TMZ had no effect on SERCA2a expression while they significantly increased the phosphorylation of PLB at only Ser16 and at both Ser16 and Thr17 sites, which is responsible for regulating SERCA2a activity. In contrast, the downregulation of CaMK II expression indicated that the enhanced phosphorylation of PLB was mainly mediated by PKA. Correspondingly, p-PKA expression was also upregulated in the DHI- and TMZ-treated groups compared with that in the MI group. This was consistent with the phosphorylation level of PLB, indicating that PKA-dependent phosphorylation of PLB could explain the enhanced heart contraction with DHI pretreatment.

## 4. Discussions

In this study, trimetazidine and Danhong injection were compared in alleviating MI injury. Our results demonstrated that DHI and TMZ decreased myocardial infarct size, improved ultrasonic heart function, and reduced levels of CK, LDH, and AST after MI. Further RNA-seq analysis and experiments indicated that TMZ and DHI inhibited cell apoptosis by downregulating the CaMKII pathway, while they enhanced cardiac myocyte contractile ability and maintained calcium homeostasis possibly through the PKA signaling pathway.

Ca^2+^ handling was identified as a vital process for the treatment of MI. Disorders in calcium homeostasis have emerged as a leading factor in causing dysfunction and damage during myocardial infarction. During the cardiac excitation-contraction coupling process, myocyte contraction is initiated in a Ca^2+^-induced Ca^2+^ release (CICR) manner, in which a small influx of calcium ions through L-type Ca^2+^ channels (LTCCs) triggers the opening of the Ca^2+^ release channel/ryanodine receptor (RyR2) in the sarcoplasmic reticulum (SR), leading to a large Ca^2+^ release from the SR. The increase in free intracellular Ca^2+^ concentration ([Ca^2+^]_i_) promotes calcium binding to the protein troponin C to drive myofilament sliding, and then myocytes are shortened. Immediately, [Ca^2+^]_i_ decrease to allow Ca^2+^ to dissociate from troponin and the myocytes to relax. Intracellular Ca^2+^ ions are mainly removed by four calcium transporters: SR Ca^2+^-ATPase, sarcolemmal Na^+^/Ca^2+^ exchanger, sarcolemmal Ca^2+^-ATPase, or mitochondrial Ca^2+^ uniporter. In normal rat ventricular myocytes, the SR Ca^2+^-ATPase promotes the uptake of [Ca^2+^]_i_ into the SR and contributes to 92% of calcium removal from the intracellular space. However, the Na^+^/Ca^2+^ exchanger removes [Ca^2+^]i out of myocytes and only contributes to 7% while others contribute less than 1% to the decrease in [Ca^2+^]_i_ [22]. Therefore, SR Ca^2+^-ATPase plays a vital role in SR calcium loading and release, as SERCA2a activity is closely regulated by phospholamban (PLB), which has two different phosphorylation sites, specifically PKA-dependent (Ser16) and CaMK II-dependent (Thr17) phosphorylation sites.

CaMK II is a central regulator of intracellular Ca^2+^ transport and heart contraction [23]. Recent studies have indicated that excessive activation of CaMK II can result in intracellular Ca^2+^ overload and further lead to myocardial cell death, ventricular arrhythmia, and even heart failure [24, 25]. The elevation of CaMK II levels after myocardial infarction can lead to a paradox, which is beneficial to cardiac myocyte calcium release and uptake but may lead to cell apoptosis [26, 27]. Considering the multiple defects caused by excessive CaMK II activity in myocardial Ca^2+^ homeostasis, CaMK II inhibition is used to prevent myocardial injury, such as in myocardial ischemia-reperfusion, myocardial infarction, and Ca^2+^-related heart injury [24]. In this study, both TMZ and DHI decreased CaMK II and cleaved caspase-3 levels, indicating that TMZ and DHI protected against MI to reduce infarct size to maintain myocardial contractility through the inhibition of CaMK II and reduction of cell apoptosis [21]. In this study, both TMZ and DHI decreased CaMKII and cleaved caspase-3 levels, indicating that TMZ and DHI protected against MI to reduce infarct size to maintain myocardial contractility through the inhibition of CaMK II and the reduction of cell apoptosis.

According to the CaMK II paradox, myocyte contractility should be immediately reduced by the CaMK II pathway after MI. However, we found that cardiac myocyte contractility increased after DHI treatment compared with that with MI alone; thus, we hypothesized that DHI can upregulate other pathways to enhance cell shortening. One of all the major symptoms of myocardial infarction, reduced heart contractility, is accompanied by decreased SERCA2a in SR. Recent studies have indicated that enhanced SERCA2a/PLB activity could rescue myocardial contractility and improve heart function. Calcium transient data showed that calcium removal capabilities were elevated in the DHI- and TMZ-treated groups. Because calcium removal occurs mainly by SERCA2a and NCX [28], caffeine-induced calcium release assessment was performed to differentiate the SERCA2a and NCX functions. The data showed that SERCA2a activity was deregulated and NCX function was downregulated in the DHI- and TMZ-treated groups. Further western blotting data showed that PKA was upregulated in the two groups, which can phosphorylate PLB at Ser16 to upregulate SERCA activity. DHI and TMZ increased cardiac myocyte contractile function after myocardial infarction by enhancing SR calcium loading and RyR2 activity to increase sensitivity to calcium release. Upregulation of p-Ser16-PLB and p-PKA expression by DHI and TMZ indicated that the PKA pathway was involved in calcium handling in cardiac myocytes at infarcted edge. These results indicate that the surviving cells contract more robustly such that the heart pumping function is compensated and the attenuated blood supply to the other tissue due to myocardial infarction is alleviated.

On the one hand, DHI and TMZ reduced apoptosis; on the other hand, they increased cell shrinkage, and these two aspects of regulation maintained the normal function of the heart after myocardial infarction. Heart contractility and Ca^2+^ homeostasis could be improved by TMZ, consistent with previous data [29]. Importantly, this is the first demonstration that DHI can modulate Ca^2+^ homeostasis, and the effect of DHI was comparable to the effect of TMZ. Our results demonstrated that both TMZ and DHI inhibited cell apoptosis by downregulating the CaMKII pathway, while they enhanced cardiac myocyte contractile ability and maintained calcium homeostasis possibly through the PKA signaling pathway. In this study, only a little difference in modulating calcium was observed during MI between treatment of DHI and TMZ. According to Figure 5, TMZ-treated MI rats had a stronger calcium removal capability while DHI-treated MI rats caused more calcium release from the sarcoplasmic reticulum in the excitation-contraction coupling.

## 5. Conclusions

Taken together, the results in this study demonstrated that DHI and TMZ protects against MI by inhibiting cell apoptosis by inhibiting the CaMKII pathway and enhancing cardiac myocyte contractile function after myocardial infarction, possibly through the PKA signaling pathway, to enhance calcium handling capability.

## Data Availability

The data about the RNA-seq analysis was uploaded into NCBI https://dataview.ncbi.nlm.nih.gov/object/PRJNA533004, which can be downloaded after the data release. As for the other data used to support the findings of this study, they are available from the corresponding author upon request.

## Supplementary Materials

Supplementary Materials Table S1: RNA-seq data of DHI in resisting myocardial ischemia. Table S2: calcium transient and sarcomere shortening amplitude changed fold in myocytes.
