# Supplementary material for: Danhong Injection and Trimetazidine Protect Cardiomyocytes and Enhance Calcium Handling after Myocardial Infarction
Source: Evid Based Complement Alternat Med. 2021 Jan 15;2021:2480465. doi: 10.1155/2021/2480465 (PMC7822665; doi:10.1155/2021/2480465)
Supplement: Supplementary Materials — Table S1: RNA-seq data of DHI in resisting myocardial ischemia. Table S2: calcium transient and sarcomere shortening amplitude changed fold in myocytes. [file 2480465.f1.zip › Zhang.Supplementary materials/Zhang.TableS2.docx]

**Supplementary materials**

**The effect of DHI on contractile function of the isolated ventricular myocytes**

**Methods**

**1. Isolation of ventricular myocytes**

The LV myocytes from normal SD rats (300±20g) were isolated enzymatically as previously described. In brief, after SD rat was anesthetized, the heart was removed via sternotomy and placed on the Langendroff perfusion aortic cannula. The heart was retrogradely perfused for 5 min with oxygenated (100% O2) NT solution (normal Tyrode in mM: 137.0 NaCl, 1.2 NaH_2_PO_4_, 5.0 KCl, 1.2 MgCl_2_, 10.0 HEPES, 10 glucose, 1.2 CaCl_2_ (pH 7.4). And then, after the perfusate was switched to Ca^2+^-free Tyrode solution for 5min, followed by perfusion about 25 minutes with the same solution containing 30uM CaCl_2_ and 0.6g/ml of type Ⅱ collagenase (Worthington Biochemical). Next, the left ventricular tissue was removed and minced in KB solution ((in mM: KOH, 120; MgCl_2_, 5; L-Glutamic, 120; Taurine, 20; HEPEs, 10; EGTA, 1; D-Glucose, 10; pH 7.3 with KOH).) to obtain single myocytes. The cells are being filtered through a nylon mesh and resuspended in centrifuge tubes for centrifugation (400 rpm for 30 sec) and the supernatant was then discarded. The myocytes were resuspended and slowly added extracellular Ca^2+^ back up to 1.2 mM. The calcium-tolerant and rod-shaped cells showing clear cross striations were used for Ca^2+^ and sarcomere shortening measurements.

**2. Cytosolic Ca^2+^ transient and sarcomere shortening measurements**

Isolated cardiac myocytes were loaded with 2μM fura-2 AM keep in the dark for 30 min at room temperature, fluorescence and sarcomere shortening measurements were recorded with Fluorescence Measurement and Cell Dimensioning Systems (IonOptix, USA). After loading, cells were washed and resuspended for twitch in NT solution and then placed in the cell chamber, stimulated at 1HZ with a 4ms duration field electrical stimulation and superfused at room temperature. Myocytes were exposed to either a 340- or 380-nm wavelength excitation light. The emitted fluorescence is detected at 510 nm. The IonWizard software (IonOptix) was used to synchronously record fluorescence and sarcomere length in myocytes. Only stably contracted myocytes were administered 4μL/mL Danhong injection (DHI) for 6min. Because of the autofluorescence of DHI at 340- or 380-nm wavelength excitation, detect the background fluorescence for individual myocytes by moving the view to a nearby blank area respectively before and after DHI perfusing. The IonWizard can correct Fura 2 ratio by subtracting background and analyze the calcium transients and sarcomere shortening. The amplitude changes of contraction and calcium transient were analyzed between the before and after DHI administration.

**3. Statistical analysis**

The data obtained from the experiments were analyzed using the SPSS V17.0 software (Paired-sample t-test) and the results are shown as the mean  ± Standard Deviation (SD) with significant p values set as ^*^P < 0.05 versus basal.

**Results**

To investigate the effect of DHI on the isolated left ventricular myocytes, this study also analyzed the amplitude changes of contraction and calcium transient between the before and after drug administration. As indicated in the table, it demonstrated that DHI had not any effect on the myocytes at the 4μL/mL concentration.

Table S2. Calcium transient and sarcomere shortening amplitude changed fold in myocytes

|  | Amplitude fold  DHI 4μL/mL (n=8) |
| --- | --- |
| Calcium transients |  |
| Baseline | 0.977±0.039 |
| Departure velocity | 0.936±0.131 |
| Peak height | 0.946±0.054 |
| Time to peak | 1.037±0.074 |
| Time to 50% peak | 1.040±0.151 |
| Return velocity | 0.950±0.132 |
| [Ca^2+^]_i_ tau （decay time constant） | 0.988±0.037 |
| Sarcomere shortening |  |
| Baseline | 0.995±0.005 |
| Departure velocitys | 0.974±0.177 |
| Fractional shortening | 0.975±0.144 |
| Time to peak | 1.012±0.040 |
| Time to 50% peak | 1.021±0.064 |
| Return velocity | 0.978±0.203 |
| Time to 50% relaxation | 1.003±0.063 |

Values are means ± SD. [Ca^2+^]_i_, cytostolic Ca^2+^ concentration.
